# Editorial: Chocolate and Health: Friend or Foe?

**DOI:** 10.3389/fnut.2017.00067

**Published:** 2017-12-22

**Authors:** Mauro Serafini, Emilio Jirillo

**Affiliations:** ^1^Functional Food and Metabolic Stress Prevention Laboratory, Faculty of Biosciences and Technologies for Agriculture, Food and Environment, University of Teramo, Teramo, Italy; ^2^Department of Basic Medical Sciences, Neuroscience and Sensory Organs, University of Bari, Bari, Italy

**Keywords:** cocoa, chocolate, humans, antioxidants, immunity, inflammation, blood pressure, flavonoids

**Editorial on the Research Topic**

**Chocolate and Health: Friend or Foe?**

In the ancient past, cocoa has been appreciated as a high-calorie food to boost energy in soldiers and for its undefined medicinal and mystical properties, granting to chocolate the forbidden title of “food of God,” primordial gift for the health of mankind. Later on, as a common place, the overall perception of chocolate consumers was that of a “charming” and appealing food with the negative aspects related to high caloric content, ultimately, leading to consider chocolate as “junk food” for its obesogenic calories.

In the last years, in view of the renewed interest for nutrition science, in search of alternative sources of health-promoting foods and ingredients, a large body of research has been conducted to unravel the pros and cons of cocoa consumption in relation to human health. The supposed protective action of cocoa seems to be multifactorial, also involving different aspects of the functional arrays of physiological defenses of the body, and, in particular, the immune system. The major aim of this Research Topic (RT) is, therefore, to provide the reader with an objective picture of the state of art on the association between cocoa consumption and health. Special emphasis will be placed mainly on human trials, encompassing the effects on antioxidants of chocolate manufacturing processes, on the one hand, and, on the other hand, the evaluation of putative healthy activities exerted by both chocolate and cocoa, with special reference to immune responsiveness, cardiovascular function, and cognitive behavior.

The RT starts focusing on the effects of technology processes on the *in vitro* antioxidant activity of chocolate (De Mattia et al.). This paper provides a very informative figure on the impact of each processing steps on the antioxidant content from raw bean to conched chocolate, highlighting the massive loss of redox ingredients during the food chain. De Mattia et al. reviewing the body of evidence about the antioxidant role of chocolate in long-term intervention trials, highlight the lack of studies on the effects of processing *in vivo*, also suggesting the importance of optimizing technological process linked with more pieces of evidence from human studies. This is in order to advice consumers about the “optimal” dose of chocolate.

Strengthening the findings by De Mattia et al. about the importance of testing chocolate in subjects characterized by an ongoing oxidative stress rather than in “not stressed” subjects, Ioannone et al. clearly show that chocolate polyphenol extract was more effective in inhibiting oxidative burst in human neutrophils and monocytes isolated from obese and overweight subjects, characterized by a more enhanced oxidative/inflammatory stress, than that observed in lean subjects. This work suggests the potential role of cocoa and chocolate’s ingredients to modulate, through a redox mechanism, a key aspect of the cell-mediated immune response. In this direction, the manuscript from Camps-Bossacoma et al. expands such a perspective, reviewing the role of cocoa as a dietary modulator of the immune system, mainly in terms of antibody response, at systemic and mucosal level, as well as of cytokine release and receptor expression. Results document that the effects of cocoa are exerted at multiple steps, from antigenic presentation and cytokine production by T helper cells to the intestinal homing of activated cells, thus, providing evidence for the ability of cocoa to play a role as immune-modulator. The last part of the RT discusses the role of cocoa on cardiovascular and cognitive functions, central nervous system, and gut health. Ludovici et al. provide a comprehensive overview on interventional studies in humans looking at the effects of chocolate on blood pressure and endothelial function. A detailed and clear picture of the mechanisms of action of cocoa’s flavonols in improving markers of cardiovascular function and of all the variables involved is provided in figure 1. In their conclusions, authors comment on the importance of proper technological process (in agreement with De Mattia et al.) to have a high content of flavonoids in commercially available chocolate in order to maximize the cardiovascular benefit minimizing sugar and energy content. The fascinating and still unclear role of chocolate in neuromodulation and neuroprotective actions in humans is critically discussed by Socci et al. suggesting an array of potentiality for chocolate in protecting human cognition and counteracting cognitive decline in age-related neurological disorders. The manuscript by Magrone et al. discusses through a broad overview the different mechanisms of actions of cocoa’s polyphenols, involving cellular transcription factors, specific kinases and signal transduction pathways from biological setting to clinical applications such as vascular and neurological dysfunctions during aging, obesity, and neurological disorders. Finally, the manuscript of the RT (Petyaev and Bashmakov) highlights the need to establish and improve a strict and fruitful connection between food industry and medical sciences to fill up certain gaps such as the absence of clinically justified recommendations.

To summarize, the collection of review and research articles presented under the RT provide a comprehensive set of information on the importance of cocoa and chocolate as a functional food able to modulate different aspects of human’s physiological response to stress, such as immunity, and to optimize cardiovascular and cognitive functions. Despite many scientific efforts, the “optimal” dose of cocoa sufficient to display a protective effect is still object of debate. One can envisage that the extremely high content of bioactive ingredients makes conceivable a functional effect at low doses (around 5–7 g/day) without affecting much the daily caloric intake. We hope that this RT will prompt a critical “thinking” in the context of the scientific community on the association between chocolate and health, providing clues for further research developments.

## Author Contributions

The authors confirm being the only contributors of this work and approved it for publication.

## Conflict of Interest Statement

The authors declare that the research was conducted in the absence of any commercial or financial relationships that could be construed as a potential conflict of interest.

